# Large Scale Synthesis of Carbon Dots and Their Applications: A Review

**DOI:** 10.3390/molecules30040774

**Published:** 2025-02-07

**Authors:** Zhujun Huang, Lili Ren

**Affiliations:** School of Chemistry & Chemical Engineering, Southeast University, Nanjing 211189, China; 213183525@aa.seu.edu.cn

**Keywords:** carbon dots, large-scale fabrication, application

## Abstract

Carbon dots (CDs), a versatile class of fluorescent carbon-based nanomaterials, have attracted widespread attention due to their exceptional optical properties, biocompatibility, and cost-effectiveness. Their applications span biomedicine, optoelectronics, and smart food packaging, yet large-scale synthesis remains a significant challenge. This review categorizes large-scale synthesis methods into liquid-phase (hydrothermal/solvothermal, microwave-assisted, magnetic hyperthermia, aldol condensation polymerization), gas-phase (plasma synthesis), solid-phase (pyrolysis, oxidation/carbonization, ball milling), and emerging techniques (microfluidic, ultrasonic, molten-salt). Notably, microwave-assisted and solid-state synthesis methods show promise for industrial production due to their scalability and efficiency. Despite these advances, challenges persist in optimizing synthesis reproducibility, reducing energy consumption, and developing purification methods and quality control strategies. Addressing these issues will be critical for transitioning CDs from laboratory research to real-world applications.

## 1. Introduction

Carbon dots (CDs) represent a novel type of fluorescent carbon-based nanomaterials, characterized by a size smaller than 10 nm and a surface abundant in nitrogen and oxygen functional groups [1]. Since their initial discovery in 2004 by Xu et al. [2], these materials have garnered significant attention owing to their distinctive optical properties, low-toxicity, excellent biocompatibility, and facile functionalization [3,4,5]. Valcarcel’s group categorized CDs into graphene quantum dots (GQDs), carbon quantum dots (CQDs), and carbon nanodots (CNDs) based on their properties, crystal structure, and quantum confinement effects [6]. According to the comprehensive analysis of the structural and performance characteristics of CDs, Xia et al. defined the carbon dots with a polymer/carbon hybrid structure, arising from incomplete carbonization, as carbonized polymer dots (CPDs) [7].

CDs are typically synthesized from biocompatible carbon sources, such as natural sugars and amino acids, exhibiting minimal cytotoxicity and excellent biocompatibility. Recent studies have demonstrated that these CDs maintain high cell viability (>80%) and are efficiently excreted through renal clearance within 24 h without significant organ accumulation, reinforcing their potential for biomedical applications [8,9]. As a result, CDs have been widely explored for biomedical imaging [10], drug delivery [11,12], and in vivo diagnostics [13,14], serving as superior alternatives to conventional fluorescent materials. Additionally, the surface of CDs is rich in functional groups (e.g., carboxyl, hydroxyl, amino groups), which facilitate doping and surface modifications. These modifications significantly enhance the optical properties of CDs, including their high photoluminescence quantum yield (PLQY) [15], tunable emission wavelengths [16], and excellent photostability [17]. As a result, CDs hold substantial promise in diverse fields, including fluorescence sensing [18], optical imaging [19], and optoelectronic devices [14]. Furthermore, CDs exhibit a high specific surface area, superior electron-transfer efficiency, numerous edge defects, and photoinduced redox properties [20]. These features make them particularly attractive for spectroscopic uses, especially in energy-related fields like photocatalysis [21,22,23], light-emitting diodes (LEDs) [24,25], energy storage (e.g., photovoltaic cells [26,27], and lithium-ion batteries [28,29]). However, to facilitate the practical application of CDs in these fields, achieving large-scale production is crucial.

**Figure 1:**
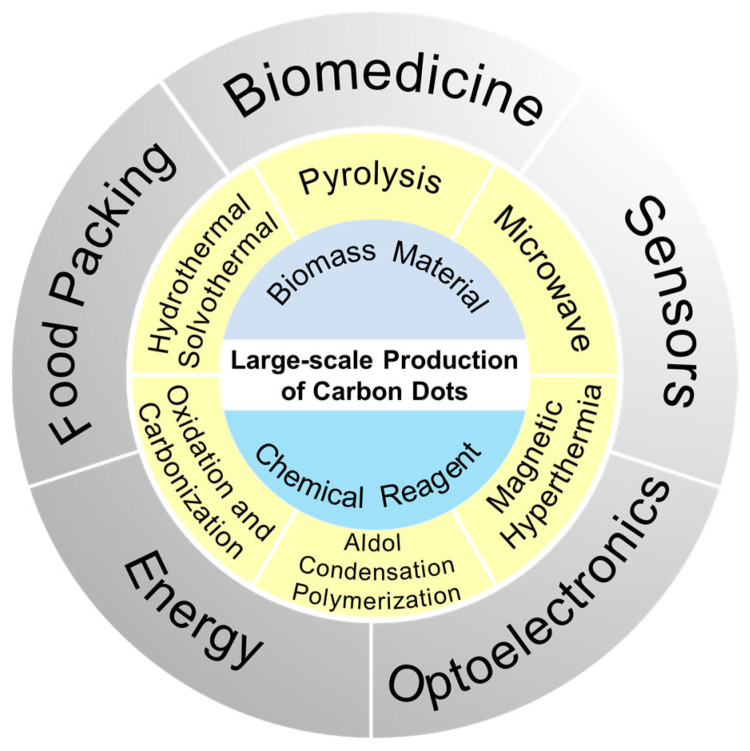
Outline of synthesis and application of CDs.

Currently, research on large-scale synthesis primarily focuses on the selection of raw materials and the optimization of synthesis techniques. In terms of raw materials, biomass resources (e.g., natural resources [30,31,32,33,34], agricultural waste [35,36,37], by-products from the food industry [38,39,40]) and industrial by-products (e.g., coal tar [41,42,43] and petrochemical waste [44,45,46]) are ideal carbon sources due to their wide availability, low cost, and alignment with the principles of green and sustainable development. Regarding synthesis methods, ambient temperature and pressure synthesis (e.g., aldol condensation and molten-salt methods [47,48,49]) and synthesis in open systems can reduce energy consumption and equipment demands. Furthermore, one-step synthesis [50,51,52] simplifies the process by integrating carbonization and functionalization in a single step, thus avoiding the complexities and resource wastage associated with multi-step reactions. Additionally, green processes such as solvent-free pyrolysis [53,54,55] achieve reactions in the solid-state, thereby enhancing safety and environmental sustainability. The optimal integration of these raw materials and synthesis methods facilitates efficient, cost-effective, and sustainable large-scale production of CDs, providing a solid foundation for their practical applications across various domains. In light of this, this mini-review commences with an examination of the synthesis methods, reviewing the large-scale preparation techniques and research advancements that have enabled the production of CDs at the gram-scale in recent years (Figure 1). These methods include pyrolysis, hydrothermal/solvothermal processes, microwave-assisted synthesis, magnetic hyperthermia, aldol condensation polymerization, oxidation, and others, alongside a brief analysis of the associated raw materials.

## 2. Synthesis Methods

According to the distinct carbon sources, the synthesis pathways of CDs can be broadly categorized into the “top-down” and “bottom-up” approach. The top-down method involves the physical or chemical stripping process from bulk carbon materials (e.g., graphite [56], carbon fibers [57], and carbon black [58]) to nanoscale CDs. While most techniques can achieve this within a relatively brief time, they necessitate specialized physical or chemical apparatus, including lasers, electrochemical reactors, and ball mills. Conversely, the bottom-up method entails the gradual carbonization and polymerization of small molecules (e.g., citric acid, folic acid, urea, and phenylenediamine) or polymer precursors through chemical reactions. This synthesis process is characterized by its simplicity, cost-effectiveness, and scalability, making it the preferred choice for large-scale production of CDs. Overall, selecting the appropriate synthesis method depends on the desired characteristics of the CDs, the intended production scale, and economic considerations.

### 2.1. Pyrolysis Method

**Figure 2:**
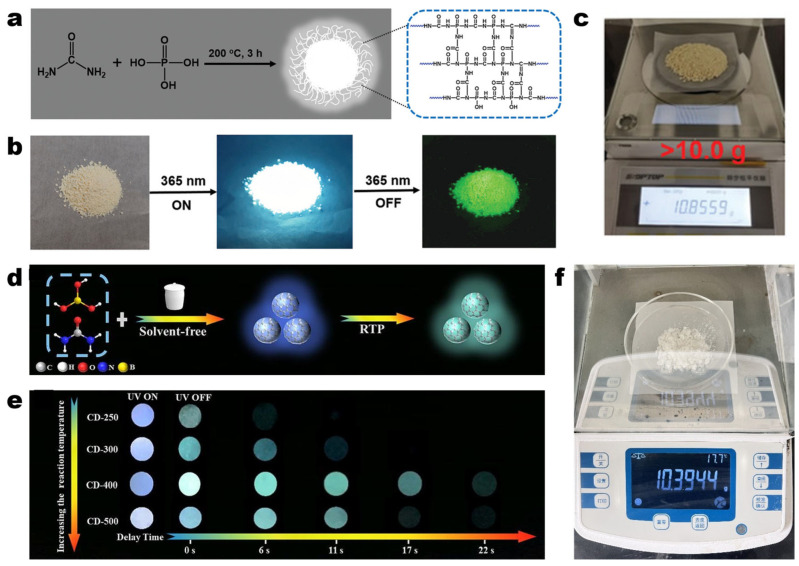
(**a**) Schematic illustration of preparation of SW-CPDs [62]; (**b**) Photographs showing RTP behavior of SW-CPDs under 365 nm UV light [62]; (**c**) SW-CPDs obtained in a single reaction with weight exceeding 10 g [62]; (**d**) Schematic illustration of preparation of URTP CDs [64]; (**e**) Photographs showing RTP behavior of URTP CDs under 365 nm UV light for CD-250, CD-300, CD-400 and CD-500 [64]; (**f**) Photograph of CD-400 in large-scale synthesis [64].

Conventional pyrolysis is a method that uses high temperatures (typically between 300 and 1000 °C) under an oxygen-free or inert atmosphere to thermally decompose carbon source materials, forming CDs [59,60]. During this process, organic molecules undergo carbonization and cracking, resulting in carbon cores along with the formation of surface functional groups. For instance, Wang et al. proposed a vacuum-heating synthesis method in which phosphoric acid (PA) and ethylenediamine (EDA) were mixed in water and directly heated to 250 °C under vacuum conditions. After the reaction was completed, 11.5 g of multiple-atom co-doped blue-emitting CDs were obtained through purification and drying, exhibiting room temperature phosphorescence (RTP). The oxygen-free environment in the vacuum enhanced the carbonization level of the CDs while preventing surface oxidation. Comparative experiments showed that this method significantly improves the optical properties of the CDs [61]. PA is frequently employed as a phosphorus source and functional reagent during the synthesis of CDs. Throughout the synthesis process, PA acts as an acidic catalyst, facilitating the carbonization reaction of the precursor material. This not only leads to the formation of an ordered π-conjugated structure but also introduces a heavy atom effect, thereby promoting triplet state formation and RTP emission. As shown in Figure 2a–c, Wang et al. utilized urea and PA as precursors and subjected them to heating at 200 °C for a duration of 3 h, resulting in the production of over 10 g of CPDs (81% yield). The synthesized CDs exhibited a RTP lifetime of 7 s [62]. Urea decomposes and releases nitrogen at relatively low temperatures (150–250 °C), making it an excellent nitrogen source during synthesis. This enables effective nitrogen doping, which enhances the electronic structure and fluorescence properties of CDs. Ren et al. [63] and Jin et al. [64] used this as raw material to prepare high-yield CDs (105 g and 10 g, respectively) with different synthesis strategies. Specifically, Jin et al. employed a solvent-free approach (Figure 2d) to blend urea with an excess of boric acid (BA) powder. Following pyrolysis at 400 °C for 5 h, over 10 g of CDs (Figure 2f) were successfully synthesized. Additionally, the surplus BA in the precursor material formed a B_2_O_3_ layer on the surface of the CDs, significantly enhancing their rigidity and conferring excellent stability to the modified CDs. This also promotes the RTP effect (Figure 2e) [64]. The absence of a liquid phase in the synthesis process eliminates the need for subsequent purification and drying steps, thereby making this method highly suitable for large-scale production. Bai et al. successfully synthesized solid fluorescent CDs via a solvent-free method. By utilizing o-phenylenediamine (o-PDA) as the carbon source and aluminum chloride hexahydrate (AlCl_3_·6H_2_O) as the catalytic assistant, they were able to prepare 13.98 g of solid blue fluorescent CDs, which also demonstrated RTP behavior [65]. Another solid-phase synthesis strategy that does not necessitate solvent involvement is the molten-salt method. Li et al. ground and heated citric acid (CA) with molten salt at 220 °C for 6 h, subsequently obtaining 1.98 g of CDs (CAs-CDs) with a yield of 39.6% after purification. To demonstrate the versatility and scalability of the molten-salt method, they used various precursors to synthesize CDs through the same procedure. The optical characteristics of the newly synthesized carbon dots were comparable to those of CAs-CDs. Notably, when sodium lignosulfonate was utilized as the carbon source, the yield of carbon dots (SL-CDs) reached an impressive 66.7%, highlighting the significant potential of this method for large-scale CDs production [66].

### 2.2. Hydrothermal and Solvothermal Method

**Figure 3:**
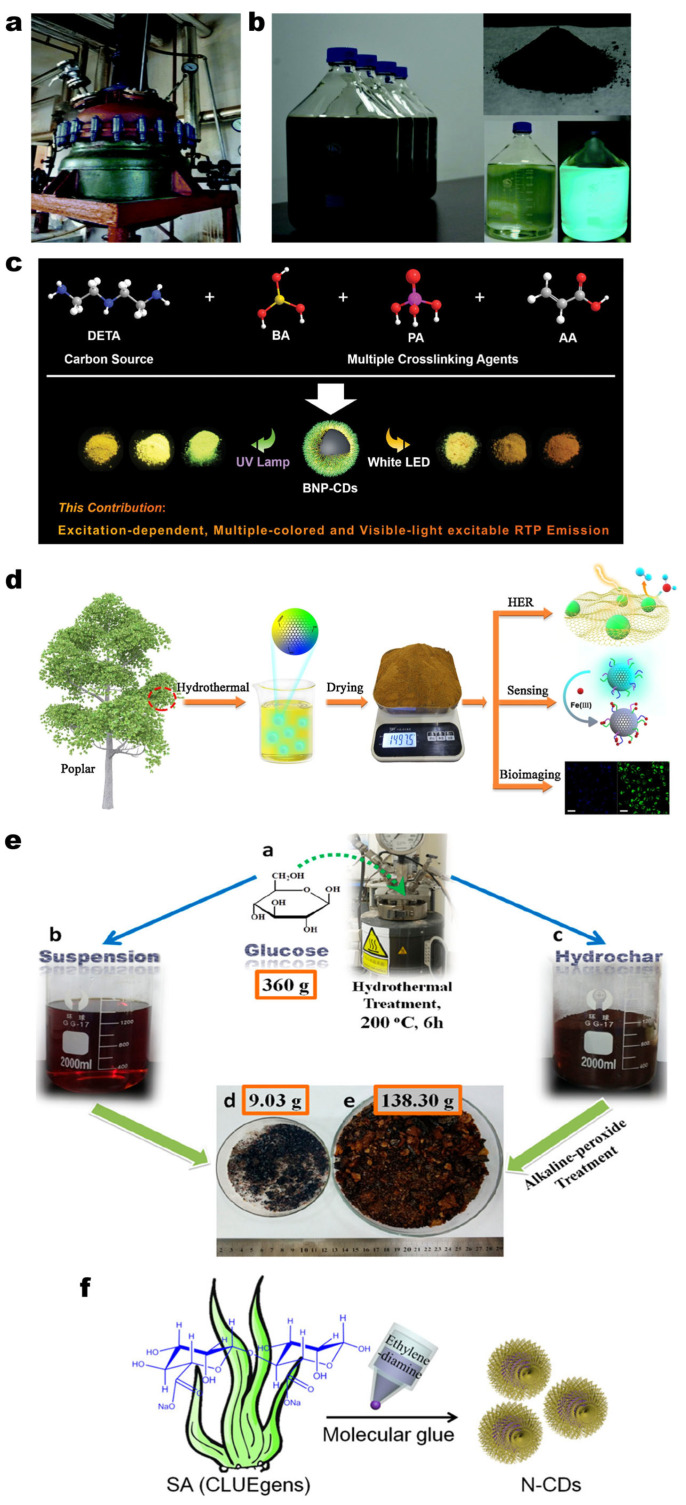
(**a**) Photograph of industrial-level apparatus (50 L stainless steel autoclave) [71]; (**b**) Photograph of concentrated aqueous dispersions (20 L) of GQDs available on an industrial scale, dried powder (100 g) and a diluted dispersion under a UV light [71]; (**c**) Schematic illustration of preparation of BNP-CDs by utilizing various crosslinking agents and heteroatoms co-doping [75]; (**d**) Schematic illustration of synthesis of CDs from poplar leaves [78]; (**e**) Schematic illustration of hydrothermal synthesis process of CDs, followed by alkaline treatment. The original labels (**a**–**e**) correspond to different stages in the referenced study [79]; (**f**) Schematic illustration of preparation of N-CDs from sodium alginate [80].

The hydrothermal method involves the use of a sealed reactor where a carbon source is heated in an aqueous solution, leading to its decomposition and carbonization under relative high temperatures and self-generated pressures, ultimately forming CDs [67]. The solvothermal method is similar to the hydrothermal method, however, it employs an organic solvent instead of water. Both of the two methods are widely used by researchers, because the size and surface characteristics of CDs can be modified by altering the temperature, reaction duration, and precursor selection. Numerous studies have reported the synthesis of high-yield CDs via the hydrothermal method, demonstrating that various raw materials possess the potential for large-scale production of CDs [68,69]. For instance, Wang et al. utilized 1,3,6-trinitropyrene as a carbon source, and achieved 1.26 g (63% yield) of green-emissive GQDs powder following nitration and subsequent hydrothermal treatment in an alkaline aqueous solution (200 °C, 10 h) [70]. Subsequently, they refined the process by substituting nitration with sulfonation and scaling the reaction to industrial-level apparatus (Figure 3a). Specifically, 1,3,6-trinitropyrene was dispersed in sodium sulfite, stirred for 30 min, and then heated in an autoclave at 130 °C for 12 h. This resulted in approximately 100 g of green fluorescent-emitting GQDs with 42% quantum yield (Figure 3b). Upon evaluation, these GQDs exhibited superior optical stability, thermal stability, colloidal stability, and biocompatibility, indicating significant potential for large-scale production [71]. Next are some examples of solvothermal synthesis. In 2017, Ding et al. prepared a formamide solution containing CA and EDA, which was subsequently heated in a reactor at 180 °C for 4 h. After filtration and purification, approximately one gram of red fluorescent CDs was obtained. Because red fluorescence can penetrate the animal body and overcome the interference of the animal tissue’s own fluorescence, the large-scale synthesis of such high-performance red CDs (with a fluorescence emission peak at 627 nm and a PLQY as high as 53%) holds significant implications for the biomedical field [72]. Wang et al. synthesized orange–red fluorescent CDs by using 1,8-diaminonaphthalene and phthalic acid as precursors and N,N-dimethylformamide (DMF) as the solvent, reacted at 200 °C for 6 h to obtain 1.0326 g orange–red fluorescent CDs powder (yield 79.6%). It was observed that the fluorescence of the CDs is solvent-dependent, with the emission color changing according to the solvent used. What is even more remarkable, this change was independent of the excitation wavelength. It is found that this change was not only related to the polarity of the solvent but also related to the formation of hydrogen bonds between the carbon surface and the solvent. Leveraging this optical property, multi-colored LEDs can be readily fabricated [73].

Serving as a source of boron, BA can be incorporated into the structure of CDs. The introduction of boron-containing functional groups onto the surface of CDs facilitates their binding with other molecules, thereby enhancing their application performance in biosensing, catalysis, and drug delivery. Furthermore, BA is cost-effective and readily available, exhibiting high reactivity under hydrothermal or solvothermal conditions, which enables effective boron doping. In 2020, Zhang et al. synthesized nitrogen–boron doped carbon dots (N,B-co-CDs) by mixing BA, L-tartaric acid, and L-arginine in an aqueous solution, followed by a reaction at 180 °C for 10 h. After filtration and purification, 1.2703 g of N,B-co-CDs were obtained, with a yield of 21.3%. The yield of N,B-CDs was notably higher within the pH range of 5–10 compared to other conditions. These N,B-CDs exhibit excellent fluorescence stability and hold significant potential for the detection of [Fe^3+^] and *Escherichia coli* [74]. As shown in Figure 3c, Wu et al. utilized diethylenetriamine (DETA) as a carbon source, while BA, PA, and acrylic acid (AA) served as crosslinking agents to synthesize a kind of excitation-dependent phosphorescent CD (BNP-CDs). Furthermore, they successfully scaled up the experiment to produce 141 g of BNP-CDs from 442 g of raw materials under reaction conditions of 200 °C for 12 h. The products obtained from the scaled-up reaction were nearly identical to those produced at the laboratory scale (4.42 g of raw materials), thereby demonstrating the feasibility of large-scale production [75]. Yao et al. successfully synthesized a series of color-tunable, gram-scale CDs (as detailed in Table 1) using tartaric acid as the carbon source, and arginine and BA as nitrogen–boron co-dopants, by varying the reaction temperature. The synergistic effect of nitrogen–boron co-doping effectively mitigated the quenching phenomenon induced by CD aggregation, thereby enhancing the application potential of these CDs in the LED industry [76].

A wide range of biomass materials can be utilized. In 2015, Zhang et al. synthesized 3 g of blue CDs (30% yield) from bee pollen at 180 °C for 24 h. The commercial bee pollen used cost approximately $0.011 per gram, making it both inexpensive and readily available, thus significantly enhancing its potential for large-scale production [77]. In 2019, Li et al. produced 1497.5 g of CDs from poplar leaves (Figure 3d), which exhibited excellent properties (long-term stability, photostability, and low toxicity). They also explored the potential applications of these CDs in various fields, including catalysts for hydrogen evolution reactions, metal ion sensors, and fluorescent probes for bioimaging [78]. In addition to the direct treatment of biomass materials, significant achievements have been made in the research of certain biomass-derived carbon sources. Jing et al. heat-treated 360 g of glucose at 200 °C for 6 h to obtain a solid–liquid mixture. After solid–liquid separation of the resulting product, 9.03 g of CDs were obtained from the suspension through conventional purification. By applying NaOH/H_2_O_2_ peroxide treatment to the solid by-product (hydrochar), an additional 138.3 g of CDs were recovered (Figure 3e). This method enables a more comprehensive utilization of the products generated by the one-pot hydrothermal process, thereby improving the raw material utilization rate, reducing costs (the cost of 1 g of CDs is approximately $6), and making it suitable for large-scale production [79]. However, the drawbacks associated with using this method to acquire CDs are also evident. Specifically, the purification process is excessively complex and time-consuming, a challenge that remains prevalent when utilizing biomass or biomass-derived materials as precursors for the hydrothermal synthesis of CDs. In 2019, Wang et al. developed a novel approach for this purpose and named it the “molecular glue strategy”. The name came from the discovery that the sodium alginate molecular chain, or intramolecular carboxyl sodium group, was cross-linked by EDA through amination reactions, just like “glue” reactions. First, they dissolved sodium alginate and EDA in water, then transferred the fully mixed solution to a reactor and stirred it at 180 °C for 8 h (Figure 3f). Eliminating the need for separation and purification, after cooling to room temperature, the product could be freeze dried to obtain a kind of high-yield solid-state CDs (100 g of sodium alginate can obtain about 95 g of CDs) [80].

### 2.3. Microwave Method

Microwave synthesis is a method in which raw materials are directly heated and carbonized into CDs by absorbing and converting microwave radiation energy into thermal energy [81,82,83]. Microwave-accelerated heating can complete the synthesis of CDs within minutes, which is significantly faster than other conventional methods (e.g., hydrothermal or solvothermal methods, which usually take several hours). Furthermore, microwaves provide uniform energy distribution, which enable making CDs particles more uniform in size [84]. Moreover, the operation of microwave synthesis equipment is simple, and reaction conditions can be accurately controlled, offering potential for large-scale production of CDs [85,86]. In order to reduce equipment requirements and manufacturing costs, a number of researchers have also successfully explored the synthesis of CDs using household microwave ovens [87,88,89,90,91,92]. In current research on CDs preparation, microwave heating is often combined with other synthesis strategies, a technique collectively referred to as microwave-assisted synthesis. Compared to simple microwave synthesis, microwave-assisted methods can process a wider variety of precursors, including liquids, solids, and biomass. According to specific requirements for surface-functionalized CDs, microwave-assisted methods also allow the introduction of functional reagents (e.g., boron, nitrogen, phosphorus, and sulfur dopants [86,87,93,94,95,96,97]).

**Figure 4:**
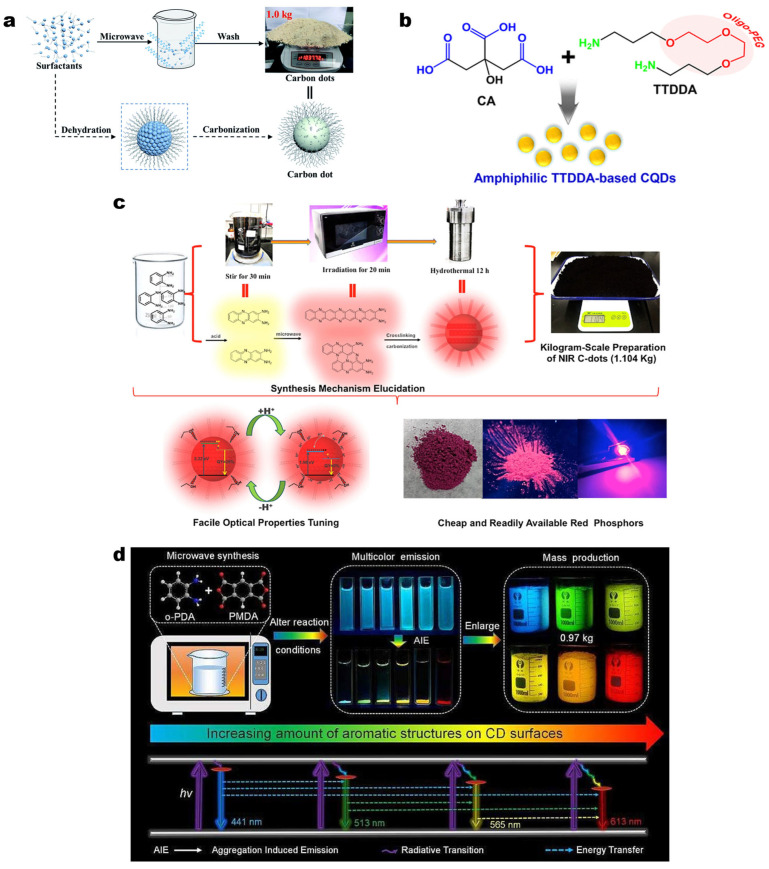
(**a**) Schematic illustration of preparation of CDs by microwave-assisted carbonization of surfactants [98]; (**b**) Illustration for the Synthesis of TTDDA-Based CQDs from CA and TTDDA [87]; (**c**) Synthesis mechanism elucidation of CDs and their applications [91]; (**d**) Schematic illustration of kilogram-scale synthesis of full-color solid-state fluorescent CDs [92].

In order to achieve industrial-scale mass production, in addition to fast, environmentally friendly, and facile synthesis methods, the availability of inexpensive and readily accessible raw materials is also an important factor to consider. Fang et al. utilized low-cost industrial surfactants (Span 40, octadecyl sucrose, soybean lecithin, and dodecyl betaine) as raw materials and successfully fabricated blue fluorescent CDs at a kilogram scale (Figure 4a). Taking CDsp (with Span 40 as the carbon source) as an example, CDsp was synthesized through a reaction under a microwave power of 1000 W for 18 min, obtaining 1037.72 g of product with a yield of 75% [98]. Apart from inexpensive and high-volume industrial products, biomass is noted for its green environmentally friendly qualities and wide availability, thereby becoming a better choice for carbon sources of CDs [99]. Calamansi is a type of biomass that can be found in several regions worldwide, and its juice (CJ, Calamansi juice) has a rich content of CA. In 2017, Regina et al. employed CA and CJ as carbon sources in combination with the passivating agent EDA though repetitive heating by microwave to prepare the CDs. By controlling the amine utilized, microwave power, the concentration and ratio of precursors, and the pyrolysis time, they identified the optimal synthesis conditions for CDs, accomplished the gram-scale synthesis of both CA-CDs (9.93 g, 99% yield) and CJ-CDs (7 g, ~7% yield) [86]. The surface modification of CDs by using an amine-rich passivation agent is a common method for enhancing fluorescence properties, hydrophilicity, and biocompatibility. In the same year, Choi et al. used 4,7,10-trioxa-1,13-tridecanediamine and CA as the precursors to obtain a type of gram-scale CDs (6.56 g, 66.4% yield) in a household microwave oven within 5 min by the microwave-assisted pyrolysis method (Figure 4b) [87].

In numerous studies, CA has been verified to be an excellent choice of carbon source for CDs [100,101,102], and phenylenediamine (PD), which possesses highly active amino groups, is also a common raw material for the synthesis of CDs. CDs synthesized with PD as the carbon source frequently exhibit distinctive optical properties (e.g., long-wave emission and multicolor emission) [16,103]. Ji et al. used microwave–hydrothermal combined treatment to prepare NIR CDs with o-PDA as the raw material (Figure 4c). They explored the mass production of CDs and dissolved o-PDA, 3-chloroperbenzoic acid (57.5 g), and H_2_SO_4_ in water. They heated the mixture in a household microwave oven at 200W for 20 min and then transferred it to an autoclave where it was heated at 200 °C for 12 h. The final mixture could be purified by simple filtration after being pH-adjusted with NaOH solution [91]. In 2023, Ding et al. dissolved PMDA (1,2,4,5-Benzenetetracarboxylic anhydride) and o-PDA in an ethanol aqueous solution. By varying the amounts of o-PDA and the reaction times, a series of kilogram-scale CDs of B-, G-, Y-, and R-CDs were synthesized via a one-step microwave-assisted method in a household microwave oven (See Table 1 and Figure 4d for more details). This method features simple equipment requirements, a high product yield, a reaction time of no more than half an hour, and the subsequent treatment merely requires centrifugation, filtration and drying operations to obtain the products, which is highly suitable for large-scale production requirements [92].

The CD optical properties of RTP have attracted the attention of many researchers, so how to facilitate the ISC process and stabilize the excitation triplet to suppress the non-radiative transition has become a key problem [104,105]. Nitrogen and phosphorus elements co-doping can facilitate the n-π* transitions to achieve triplet exciton, they can also contribute to constructing CDs’ cross-linked structure. In this case, Jiang et al. used ethanolamine and PA as the precursors to produce the gram-scale CDs (2.8 g) by microwave-assisted heating, with a reaction time of only five minutes [97]. Su et al. also used PA as the source of phosphorus element, polyethyleneimine as the source of carbon and nitrogen, and successfully prepared 1.06 g of cross-linked CDs with RTP properties by rapid microwave heating (only 30 s) [106].

### 2.4. Magnetic Hyperthermia Method

The Magnetic Hyperthermia Method (MHT) represents a technique for heating reactants through the heat generated by magnetic nanoparticles when exposed to an alternating magnetic field. The process involves the following steps: (1) mixing a carbon precursor (e.g., citrate [107], PEG [108], biomass [109]) with magnetic nanoparticles (e.g., ferrite Fe_3_O_4_); (2) under an alternating magnetic field, the magnetic particles rapidly generate heat due to hysteresis loss (primarily occurring in ferro/ferri-magnetic state), relaxation loss (mainly taking place in superparamagnetic state) and eddy current loss [110,111]; (3) the localized heat carbonizes the carbon precursor, leading to the formation of CDs.

In 2019, Chen’s group first developed MHT for the synthesis of CDs. They selected citrate (zinc citrate, sodium citrate, and potassium citrate) and carbamide as the precursors for the preparation of CDs. Ultimately, multicolor fluorescent CDs: Zn-CDs (85.3 g, 69% yield), Na-CDs (80.7 g, 64% yield), and K-CDs (74.5 g, 58% yield) were fabricated. Notably, the entire process of synthesis and separation in this study took less than an hour, which was approximately 160 times more efficient than the production of CDs using traditional strategies (e.g., hydrothermal and solvothermal) [107]. Subsequently, this group also carried out other investigations on the large-scale synthesis of CDs via MHT. For instance, 3.01 g (60.2% yield) of less toxic CDs with excitation-dependent photoluminescence (fluorescence color changed from light blue to orange) were prepared by using 5 g ammonium citrate [108]. Additionally, 25.37 g blue–green fluorescent CDs were fabricated with CA and urea as raw materials, and the mechanical properties of polymeric nanofiber films were enhanced by using the CDs [112]. Since heating can be locally implemented at the nanoscale, this method presents the advantages of high efficiency (short reaction time) and selective heating (the heating process is concentrated around the magnetic particles, avoiding the need to heat the entire system). This approach eliminates the requirement for high temperature or high pressure and reduces energy consumption. By optimizing reaction conditions and magnetic field devices, this method is anticipated to be applied in continuous flow reactors to enhance production efficiency and is suitable for large-scale production. Moreover, the follow-up treatment process of the reaction product is straightforward, and the majority of the Fe_3_O_4_ particles can be removed and recycled through the magnet, which will significantly reduce the production cost.

### 2.5. Aldol Condensation Polymerization

Aldol Condensation Polymerization is a process where carbon dots are synthesized by polymerizing carbonyl-containing molecules (such as aldehydes or ketones) with α-hydrogens through condensation reactions. Under acidic or basic conditions, deprotonation of the α-hydrogen of a carbonyl compound leads to a condensation reaction with another carbonyl compound, forming a β-hydroxy ketone intermediate. Then, the β-hydroxy ketone intermediate undergoes dehydration to form a conjugated enone, which can further polymerize to generate complex carbon chains, networks, or be carbonized into carbon cores which could crosslink to form CDs [113]. Only after the pH value of the mixed solution, obtained following the reaction, has been adjusted to a neutral state, can the solid-state CDs powder be obtained through the use of a freeze dryer. The method can utilize a wide range of carbonyl and α-hydrogen-containing precursors, such as biomass resources and sugars, which are low-cost and abundant. Furthermore, during the synthesis process, it is relatively straightforward to introduce a variety of functional groups (e.g., hydroxyl, carboxyl, and amino) due to the surface of CDs synthesized by this method being rich in branched chains and oxygen-containing functional groups, which facilitates subsequent functionalization or doping.

In 2015, Hou et al. by chance mixed acetone and sodium hydroxide without undergoing any other treatment. It was found that the mixture turned into a brown–black solid product a few days later. Through a series of analysis and characterization, the high-yield product was identified as CDs. This represents a simple and low-energy approach. What is more appealing is the high-yield production along with the simple post-processing [114]. In the next year, they used acetaldehyde solution and sodium hydroxide as the raw materials to produce a kind of large-scale CDs within a short time (vigorous magnetic stirring for 1 h), and provided a new way to fabricate phosphorus-doped carbon nanosheets [115]. Based on this work, the research team refined the preparation process in 2021, scaled up the production under ambient conditions, and successfully produced kilogram-scale CDs (1083 g) within 2 h. Furthermore, they obtained functionally doped CDs by incorporating reagents with additional functional groups during the synthesis process which would facilitate the modification of CDs [116]. Zhang et al. utilized acetaldehyde and sodium hydroxide as raw materials to synthesize 108.4 g of CDs within a two-hour timeframe through a one-step aldol condensation reaction. The CDs were subsequently dispersed in sunflower oil, which significantly enhanced the synergistic effect between the CDs and the lubricant, attributed to the rich presence of oxygen-containing functional groups on the surface of the carbon dots. Notably, the CDs demonstrated superior service life and lubrication stability under friction conditions, maintaining the friction coefficient variation of sunflower oil at nearly zero for a duration of 500 min [117]. In 2023, Gao et al. used 3-phenylpropionaldehyde and sodium hydroxide as precursors, and prepared fluorescent CDs (2.46 g) with excitation-dependent properties under atmospheric pressure. After analysis, the fluorescence properties of CDs remain stable when the pH value is 1–7, so CDs have the potential application as fluorescent probes to detect the concentration of hydrogen ions under such high acidity conditions [118]. Inspired by the Claisen–Schmidt reaction, Xu et al. utilized acetaldehyde and p-fluorobenzaldehyde as precursors to synthesize 1.88 g of yellow fluorescent fluorine-doped CDs. Subsequent testing revealed that the incorporation of fluorine atoms renders the CDs nearly insoluble in water yet maintains their solubility in various organic solvents. This modification significantly enhanced the environmental stability of the CDs-based materials without compromising their compatibility with electrolyte films [119].

### 2.6. Oxidation and Carbonization

**Figure 5:**
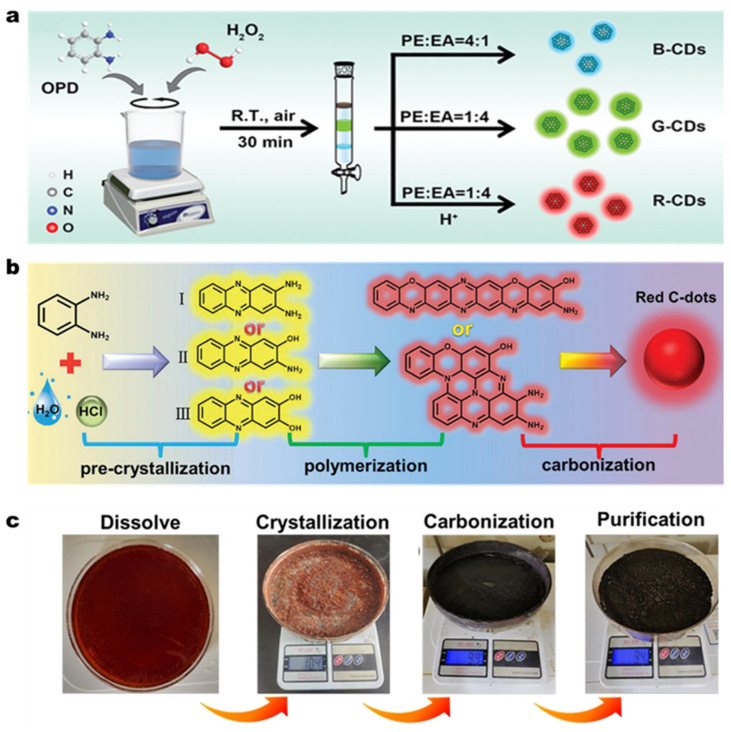
(**a**) Synthesis scheme for multi-color CDs [122]; (**b**) Schematic diagram showing the proposed formation process of CDs [125]; (**c**) Photographs of each step in the preparation of CDs [125].

Chemical oxidation involves breaking down bulk carbon sources (e.g., graphite, carbon black, or carbon nanotubes) using strong oxidizing agents (e.g., nitric acid, potassium permanganate, or persulfates) [120,121]. For example, in 2023, Ma et al. synthesized 1027.9 g of CDs utilizing o-PDA as the carbon source and hydrogen peroxide (H_2_O_2_) as the oxidizing agent in an open system. Furthermore, the crude CDs powder can be processed to yield three distinct colored (blue, green, and red) CDs through subsequent separation and treatment procedures (Figure 5a), highlighting the potential for the preparation of multi-color and white LED devices [122]. In 2024, Meng et al. also prepared high-yield (100 g) red fluorescent CDs by using o-PDA as raw material in an open system. In order to explore the mechanism of CDs formation, they divided the synthesis process into two parts: 2 h of carbon source pre-oxidation process and 10 h of acid catalysis process. Comparative experiments revealed that the optimal pH range of acid catalysis process is 5–6, achieving a high yield of 77%. It is noteworthy that the red fluorescent CDs have superior optical properties (PLQY of 33.26%), and the white light emitting diode (WLED) based on the CDs has a standard CIE color coordinate (0.33, 0.33) and a color rendering index (CRI) of up to 94.5 [123].

Carbonization usually involves thermally decomposing carbon-rich precursors (e.g., sugars, biomass, and polymers) at high temperatures to produce CDs. For instance, in 2021, Su et al. successfully synthesized 7.8 g of blue fluorescent CDs utilizing locust powder as the carbon source via the self-exothermic reaction between nitric acid and DETA within 10 min. This approach eliminates the need for an external heat source, thereby rendering the preparation process both straightforward and highly efficient [124]. Xu et al., through sustained research efforts, devised a large-scale (94 g, 94% yield) synthesis strategy for producing red fluorescent CDs with superior fluorescence properties (fluorescence emission peak at 608 nm, PLQY 25.4%) [125]. As shown in Figure 5b,c, the process involves: (1) dissolving o-PDA in an acidic solution (e.g., dilute hydrochloric acid); (2) crystallizing o-PDA under mild heating; (3) calcining the pre-crystallized o-PDA; and (4) purifying to obtain the target CDs. Under acidic conditions, o-PDA can undergo self-condensation to form 2,3-diaminophenazine (DAP). The highly π-conjugated structure of DAP facilitates the formation of sp^2^ carbon domains during carbonization, thereby enhancing the graphitization of CDs [126]. The extended conjugation in DAP leads to red-shifted emission wavelengths for the resulting CDs. Crystallization serves as an effective method to organize molecular accumulation. Pre-treatment of the CDs precursor by crystallization enables solid-phase preparation of CDs. The entire preparation process requires relatively mild conditions, thus reducing the demand on equipment and providing a promising approach for industrial-scale production of high-quality CDs.

Hu et al. developed a thermal oxidation method to synthesize nitrogen-doped CDs by integrating the advantages of two reactions. By introducing pure oxygen into a three-necked flask under heating conditions, this method facilitates the cleavage of organic molecules. The mixture of alcohol amine compounds (monoethanolamine (MEA), diethanolamine (DEA), triethanolamine (TEA)) and EDA undergoes carbonization and oxidation, leading to the rapid and efficient synthesis of CDs. It has been observed that using MEA as the precursor and reacting at a higher temperature (170 °C) and a higher oxygen flow rate (100 mL/min) for two hours is more conducive to large-scale production of uniformly sized CDs (3.36 g). Under the same heating time and oxygen flow, the yield of CDs with TEA as the precursor was significantly higher (19.12 g) [127]. To deeply understand the formation process of nitrogen-doped CDs, they continuously prepared a new type of carbon dot with MEA as the raw material. They heated 230 mL MEA and 30 g CA under air reflux at 170 °C for 10 min. After evaporative drying treatment, 39.96 g of CDs powder was obtained. Taking temperature as the node (130 °C, 150 °C, 170 °C), they extracted the products at different reaction stages for characterization. It could be inferred from the changes in particle size, absorbance and photoluminescence intensity that the production of polymers, intramolecular dehydration and shrinkage, as well as the carbonization process occurred. A number of studies in their research group have all demonstrated the superiority of MEA in synthesizing CDs. Using the same synthetic method and reaction conditions, they replaced CA with other hydrophilic carbon precursors and easily synthesized a large number of CDs within 10 min of reaction time [128] (see details in Table 1). Despite the fact that the product PLQY synthesized using alternative carbon sources did not perform as well as CA, it had been demonstrated that employing MEA as a solvent for the synthesis of CDs was a highly viable strategy for large-scale production. This method not only significantly reduced the synthesis time (10 min) but also eliminated the need for pressurized conditions during the preparation process. Furthermore, the post-synthesis treatment of the product was straightforward, involving merely the distillation of the solvent MEA and the vacuum drying of the final product.

The process of converting carbon sources into small carbon nanomaterials using strong acids (such as concentrated sulfuric acid and nitric acid) normally involves a combination of carbonization and oxidation [59,129]. The detailed steps are as follows: (1) dehydration by strong acid: When carbon sources are exposed to concentrated sulfuric acid, the acid’s strong dehydrating properties remove water molecules from the precursors, causing rapid carbonization and forming an initial carbonaceous structure. (2) Oxidative cutting: If concentrated nitric acid or a mixture of sulfuric and nitric acids is used, nitric acid acts as a strong oxidant, breaking down the carbonaceous material into smaller CDs. This process also introduces oxygen-containing functional groups (e.g., carboxyl, hydroxyl, and nitro groups). (3) Formation of CDs: Through dehydration and oxidative cutting, the carbon source is converted into uniformly sized CDs. The resulting CDs typically have surfaces rich in oxygen-containing groups, enhancing their water solubility and fluorescence properties. In 2013, Chen et al. conducted an experiment wherein they heated sucrose and oleic acid in a three-necked flask under nitrogen protection at 215 °C for a duration of five minutes. The resulting product was subsequently purified and freeze-dried, leading to the successful synthesis of 8.36 g (41.8% yield) of highly stable CDs [130]. In 2015, Liu et al. combined 1.5 g of Vulcan XC-72 carbon black with nitric acid in a three-necked flask at 135 °C for 24 h, which resulted in the successful synthesis of 1.2 g of GQDs with a yield of 75%. Notably, when the excitation wavelength ranged from 260 nm to 470 nm, the material exhibited excitation-independent photoluminescence behavior with an emission peak at 523 nm. However, as the excitation wavelength increased from 530 nm to 620 nm, the maximum emission peak position shifted to 620 nm, because of this unique optical property, it shows potential for biological applications [131]. In addition to conventional carbon sources, some researchers have opted for unconventional carbon sources through this method in their studies. For instance, in 2014, Yang et al. successfully produced high-yield CDs (60–80%, 10–120 g per batch) utilizing a kind of CNP which was obtained from Chinese ink and mixed acids as raw materials, and these CDs can be doped with heavy atoms via a simple hydrothermal reaction [132].

### 2.7. Other Methods

In addition to the aforementioned methods, numerous other reported CDs synthesis techniques have been demonstrated to achieve production scales exceeding one gram. For example, Kim et al. effectively utilized a thermal plasma jet to synthesize GQDs at a production rate of approximately 4 g per hour [133], and similarly, Ma et al. employed a micro-plasma jet to fabricate nitrogen-doped CQDs at a rate of 1 g per day, using folic acid as the carbon precursor [134]. Given that no solvents are employed, the resultant CDs typically exhibit high purity, which facilitates the purification process. Although plasma reactors can be configured as continuous-flow systems to support large-scale production, the reported yields of CDs are generally lower than those obtained through alternative large-scale production methods. In addition to plasma jets, the continuous production of CDs can also be achieved using microreactors. The microfluidic approach utilizes micro- and nanoscale channels to precisely control fluid flow and reactions, offering a highly controllable and precise method for the synthesis of CDs. Rao et al. utilized CA and EDA as precursors to successfully synthesize nitrogen-doped CDs with high PLQY using microreactors with different porosities (with foamy copper [135] or copper fibers [136]), all of which took less than 10 min. Furthermore, they conducted a systematic investigation into the impact of various conditions (including flow rate, reaction temperature, and precursor concentration) on the photoluminescence performance of the CDs [137]. The synthesis method employing CA and EDA as the precursor combination exhibits low operational costs, rapid synthesis times, and continuous production capabilities. Additionally, the utilization of minimal reagents reduces waste emissions and environmental pollution, aligning with the principles of green chemistry. There have also been reports of large-scale synthesis of CDs by top-down methods. For example, in 2014, Park et al. used ultrasonic treatment for 45 min to obtain about 120 g of CDs from 100 kg of waste food [138]. Ultrasonic treatment can achieve in situ doping in the synthesis process by adding dopants (e.g., nitrogen sources and boron sources) to improve the optical and catalytic properties of CDs. Dang et al. used polyamide resin as the carbon source, EDA as the passivating agent, and a silane coupling agent KH-570 as the co-passivating agent. After ultrasonic treatment for 3 h, white fluorescent CDs with photostability (with quantum yield of about 28.3%) were obtained [139]. Generally, the preparation of CDs via the ultrasonic method involves dissolving the precursor in water or an organic solvent, followed by placing the solution in an ultrasonic reactor. The cavitation effect induced by ultrasonic waves leads to the dehydration, polymerization, and carbonization of the precursor molecules under conditions of instantaneous high temperature and pressure, ultimately forming CDs. Compared to the conventional hydrothermal method, ultrasonic synthesis of CDs is more efficient, typically conducted at ambient to moderate temperatures, ensuring safety and convenience, which aligns with the demands for large-scale production. In addition to these, ball milling can continuously produce CDs. By subjecting the precursor material to repeated impact, shear, and friction forces in the ball mill, the particles are gradually refined into nanoscale carbon dots. This method does not require high temperatures, high pressures, or other complex reaction conditions, thereby ensuring a high level of safety and demonstrating significant potential for large-scale production [140,141,142,143].

**Table 1:** Methods for the large-scale production of CDs.

Methods	Report Years	Raw Materials	Production (g)	Production Yields (%)	Reaction Times	Reaction Temperature (°C)	Emission Peak (nm)	Quantum Yields (%)	Element Doping	Applications	Ref.
Pyrolysis	2017	CA	1.98	39.6	6 h	220	443–567	20.8	O	Bioimaging	[66]
	2020	PA, urea	10.8559	~81	3 h	200	436, 495	41	O, N, P	WLEDs	[62]
	2021	o-PDA	13.98	N/A	12	200	472	3.2	O, N	N/A	[65]
	2022	CA, urea, CaCl_2_	105.465	70	3 h	250	520	~79	O, N, Ca, Cl	Security ink	[63]
	2023	EDA, PA	11.5295	N/A	5 h	250	~420	23.1	O, N, P	Security ink	[61]
	2024	BA, urea	10.39	N/A	5 h	400	480	22.15	O, N, B	Multi-level anti-counterfeiting, digital encryption, fingerprint recognition	[64]
Hydrothermal	2014	1,3,6-trinitropyrene, sodium hydroxide	1.26	63	10 h	200	475	45	O, N, Na	Bioimaging	[70]
	2015	Bee pollen	3	30	24 h	180	425–505	6.1~12.8	N	Bioimaging	[77]
	2017	1,3,6-trinitropyrene, Na_2_SO_3_	~100	N/A	12 h	130	510	42	O, S	Bioimaging	[71]
	2019	Glucose	9.03	0.62	6 h	200	N/A	4.03	O	[Pb^2+^] Detection	[79]
	2019	Glucose, H_2_O_2_, sodium hydroxide	138.3	43.8	6 h (hydrothermal) + 8 h (alkaline-peroxide)	200 (hydrothermal)RT (alkaline-peroxide)	N/A	N/A	O	[Pb^2+^] Detection	[79]
	2019	Poplar leaves	1497.5	N/A	24 h	200	510	10.64	O, N	Bioimaging	[78]
	2019	Sodium alginate, EDA	~95	N/A	8 h	180	350–580	39	O, N, Na	Security ink, WLEDs	[80]
	2020	L-tartaric acid, L-arginine, BA	1.27	21.3	10 h	180	410–470	14.5	O, N, B	[Fe^3+^] detection, *E. coli* sensor	[74]
	2024	DETA, BA, PA, AA	141	31.9	12 h	200	475	N/A	O, N, B, P	Fingerprint collection, luminescent traffic signage, multi-level anti-counterfeiting	[75]
	2024	Tartaric acid, arginine, BA	1.133	82	10 h	180	411	34.3	O, N, B	WLEDs	[76]
	2024	Tartaric acid, arginine, BA	0.937	68	10 h	200	542	23.7	O, N, B	WLEDs	[76]
	2024	Tartaric acid, arginine, BA	1.039	75	10 h	220	660	20.5	O, N, B	WLEDs	[76]
Solvothermal	2017	CA, EDA, formamide	1	N/A	4 h	180	627	53	O, N	Bioimaging	[72]
	2024	1,8-diaminonaphthalene, phthalic acid	1.0326	79.6	6 h	200	600	23.6 (in DMF)	O, N	WLEDs	[73]
Microwave	2017	CA, EDA	9.93	99	18 min	N/A	462	1.7	O, N	N/A	[86]
	2017	CJ, EDA	~7	7	14 min	N/A	462	2	O, N	N/A	[86]
	2017	4,7,10-Trioxa-1,13-tridecanediamine, CA	6.56	66.4	5 min (700 W microwave)	N/A	440–510	29	O, N	Nanocomposite films or fibers, bioimaging	[87]
	2018	Ethanolamine, PA	2.8	N/A	5 min (750 W microwave)	N/A	417	20.52	O, N, P	Security ink	[97]
	2020	Span 40	1037.72	75	18 min (1000 W microwave)	N/A	473	0.8	O	Fluorescent nano-additives	[98]
	2021	Polyethyleneimine, PA	1.06	N/A	30 s (500 W microwave)	N/A	406	11.03	O, N, P	Security ink	[106]
	2022	o-PDA, 3-chloroperbenzoic acid, H_2_SO_4_	1104	96	20 min (200 W microwave) + 12 h (hydrothermal)	200	606	25.4	O, N	LEDs	[91]
	2023	o-PDA, PMDA, ethanol	967.20	N/A	10 min (750 W microwave)	N/A	443	55.3	O, N	Security ink, LFPs, LEDs, fingerprint collection	[92]
	2023	o-PDA, PMDA, ethanol	970.88	N/A	12 min (750 W microwave)	N/A	509	48.8	O, N	Security ink, LFPs, LEDs, fingerprint collection	[92]
	2023	o-PDA, PMDA, ethanol	971.09	N/A	16 min (750 W microwave)	N/A	563	36.7	O, N	Security ink, LFPs, LEDs, fingerprint collection	[92]
	2023	o-PDA, PMDA, ethanol	962.13	N/A	20 min (750 W microwave)	N/A	613	27.9	O, N	Security ink, LFPs, LEDs, fingerprint collection	[92]
Magnetic hyperthermia	2019	Zinc citrate, carbamide	85.3	69	N/A	230	440	55	O, N, Zn	Polymer/CDs films, nanofillers	[107]
	2019	Sodium citrate, carbamide	80.7	64	N/A	230	528	51	O, N, Na	Polymer/CDs films, nanofillers	[107]
	2019	Potassium citrate, carbamide	74.5	58	N/A	230	578	47	O, N, K	Polymer/CDs films, nanofillers	[107]
	2020	Ammonium citrate	3.01	60.2	N/A	N/A	450–600	13.5	O, N	Inkjet printing	[108]
	2021	CA, carbamide	25.37	N/A	90 s	250	500–531	67 (in ethanol)	O, N	Nanofiber films	[112]
Aldol condensation	2021	Acetaldehyde, sodium hydroxide	1083	N/A	2 h	RT	515–613	N/A	O	Carbon fibers	[116]
	2022	Acetaldehyde, sodium hydroxide, HCl	108.4	N/A	2 h	RT	N/A	N/A	O	Lubricating additive	[117]
	2023	3-phenylpropionaldehyde, sodium hydroxide	2.46	N/A	5 h	50	480	3.03	O	[H^+^] sensor	[118]
	2023	p-fluorobenzaldehyde, acetaldehyde, sodium hydroxide	1.8868	N/A	N/A	RT	597	N/A	O, F	Composite electrolytes	[119]
Carbonization	2013	Sucrose, oil acid	8.36	41.8	5 min	215	461	21.6	O	Bioimaging	[130]
	2015	XC-72 carbon black, HNO_3_	1.2	75	24 h	135	534–620	5.1	O, N	N/A	[131]
	2015	CA, MEA	39.96	N/A	10 min	170	455–523	40.3	O, N	Bioimaging	[128]
	2015	Glucose, MEA	37.23	N/A	10 min	170	457–526	13.1	N/A	N/A	[128]
	2015	Ascorbic acid, MEA	45.55	N/A	10 min	170	454–535	10.8	N/A	N/A	[128]
	2015	Cysteine, MEA	34.68	N/A	10 min	170	450–523	11.7	N/A	N/A	[128]
	2015	Glutathione, MEA	38.13	N/A	10 min	170	449–528	12.4	N/A	N/A	[128]
	2021	Locust powder, HNO_3_, DETA	7.8	N/A	<10 min	Self-exothermic	470	3.1	O, N	Chemical and temperature sensing	[124]
	2023	o-PDA, HCl	94	94	12 h	200	608	25.4	O, N, Cl	WLEDs	[125]
Carbonization/Oxidation	2014	Chinese ink, HCl, HNO_3_, H_2_SO_4_, NaClO_3_	120	80	10 h	5→15	482	6	O	N/A	[132]
Oxidation	2015	EDA, MEA, TEA	3.36 (MEA-) 19.12 (TEA-)	N/A	2 h	170 (MEA-) 270 (TEA-)	464 (MEA-)	11.9 (MEA-)	O, N	Bioimaging	[127]
	2023	o-PDA, H_2_O_2_	1027.9	N/A	30 min	RT	481 (B-CDs); 539 (G-CDs); 625 (R-CDs)	21.76 (B-CDs); 27.12 (G-CDs); 13.06 (R-CDs)	O, N	LEDs	[122]
	2024	o-PDA, acid (with a pH of 5–6)	100	77	2 h (pre-oxidation) +10 h (acid-catalysis)	60 (pre-oxidation)	603, 649, 708	33.26	O, N	WLEDs	[123]
Plasma	2014	Ethylene	4 (continuous)	10	1 h	N/A	375, 393, 406, 430	13.5	O	N/A	[133]
Ultrasonic	2014	Food-wastes, ethanol	~120	N/A	45 min (40 kHz)	RT	400–470	2.85	O	Bioimaging	[138]
	2016	Polyamide resin, EDA, KH-570 (silane coupling agent)	N/A	25.7	3 h	N/A	515	28.3	O, N, Si	WLEDs	[139]

## 3. Applications

### 3.1. Biomedical Application

**Figure 6:**
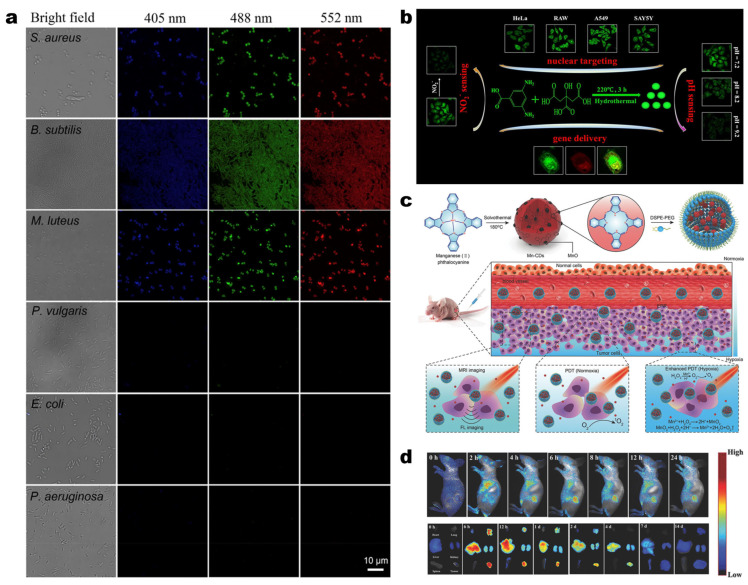
(**a**) Selective fluorescence imaging of Gram-positive bacteria using CDs [154]; (**b**) Schematic diagram of CDs preparation and applications [155]; (**c**) Schematic illustration of the Mn-CD assembly to enhance the anticancer efficiency of PDT [162]; (**d**) In vivo and ex vivo FL images of tumors at different time [162].

Numerous studies have demonstrated that CDs exhibit excellent biocompatibility, minimal toxicity or are non-toxic, and possess superior optical properties, including deep red/NIR emission [65,144,145,146,147,148,149]. Consequently, the application of CDs in the biomedical field has emerged as a prominent research direction [150,151,152,153]. In biological imaging, CDs have been extensively utilized for both in vivo and in vitro fluorescence labeling and imaging due to their tunable emission wavelengths, high photostability, and selective reactions. For instance, the gram-scale quaternized CDs prepared by Yang et al. via the solvothermal method have been demonstrated to have the ability to interact with negatively-charged bacterial cells through electrostatic and hydrophobic interactions, and exhibit selective staining effects on Gram-positive bacteria (Figure 6a) [154]. In addition, Wang et al. successfully synthesized green fluorescent carbon dots (G-CDs) with excellent nuclear targeting ability using CA and 3,5-diaminobenzoic acid as raw materials (Figure 6b). Moreover, the surface of these CDs is rich in nitrogen and oxygen functional groups, which provides the ability to bind with exogenous DNA. Therefore, they designed G-CDs as a nanocarrier for delivering DNA to the nucleolus. By observing the fluorescence image of the target cell nucleus, it was found that the exogenous DNA successfully achieved gene delivery with the help of the carrier [155]. According to the differences in the nanostructure and surface functional groups of CDs, multiple-site targeted imaging has been achieved in the reported studies (Table 2). This is of great value for monitoring the dynamic changes of living cells during the treatment process and for assisting biological analysis with fluorescence imaging, providing new ideas for targeted drug delivery and disease treatment. However, the optical properties of some CDs exhibit significant attenuation under strong light or prolonged usage. How to further improve photostability without compromising in vivo safety remains a valuable research direction.

Apart from being utilized in biological imaging, CDs possess tremendous potential as drug delivery systems due to their excellent water solubility and surface functionalization characteristics. In cancer treatment, by adjusting the surface properties of CDs (such as introducing targeting ligands [156,157,158]), they can carry drug molecules into the body and release them in a targeted manner, improving the bioavailability of drugs, enhancing the targeted therapeutic effect, and reducing side effects. Red/NIR CDs, due to their unique red/NIR absorption characteristics, possess excellent photothermal conversion efficiency and cell penetration ability, which enables them to function as photothermal agents and play an efficient role in photothermal therapy [13,159]. Meanwhile, CDs can also act as efficient photosensitizers to produce reactive oxygen species under light excitation for photodynamic therapy [160,161]. Manganese-doped CDs synthesized by Jia et al. using manganese phthalocyanine as the precursor can not only effectively generate singlet oxygen (quantum yield of 40%) but also catalyze H_2_O_2_ to further produce oxygen in an oxygen-deficient environment (Figure 6c), while simultaneously enabling dual-peak fluorescence/magnetic resonance imaging and enhanced photodynamic therapy (Figure 6d) [162]. The CDs synthesized by Cai et al. from metformin and methylene blue exhibit lysosome-targeted, light-controlled nitric oxide delivery capabilities. Additionally, these CDs demonstrate a high rate of singlet oxygen production (quantum yield of 78.36%), thereby enhancing therapeutic efficacy through the synergistic generation of nitric oxide and reactive oxygen species [163]. On the whole, by addressing challenges related to scalability, biocompatibility, and targeted precision, CDs provide a versatile and promising platform for integrated cancer therapy and diagnostics.

**Table 2:** Biomedical imaging applications of CDs.

Target Location	Report Years	Precursor	Synthesis Method	Emission Peak (nm)	Quantum Yields (%)	Applications	Ref.
Mitochondria	2018	PTCDA, PEI	hydrothermal	562	N/A	ATP fluctuation imaging	[164]
	2019	CA, m-aminophenol	microwave	520, 540, 580, 600	0.39, 0.44, 0.25, 0.40	Long-term mitochondrial imaging	[165]
	2020	CA, α-lipoic acid, urea	hydrothermal	528	11.4	Oxidative stress monitoring, mitochondria imaging	[166]
Golgi Apparatus	2017	1,3,6-trinitropyrene, Na_2_SO_3_	hydrothermal	510	42	Golgi apparatus imaging	[71]
	2017	CA, l-cysteine	pyrolysis	420	68	Long-term Golgi apparatus imaging	[167]
	2022	L-cysteine, neutral red	solvothermal	601	10.76	Golgi apparatus targeting, in vivo imaging	[168]
Nucleolus	2018	L-cysteine, m-PDA	hydrothermal	520	8.7	Nucleolus imaging, enhanced drug delivery	[169]
	2021	p-aminoazobenzene	solvothermal	605	15.1 (one-photon excitation); 18.2 (two-photon excitation)	Photodynamic therapy (PDT) and in vitro real-time dynamic monitoring of nucleolar changes	[149]
	2021	L-cysteine, m-PDA	hydrothermal	530	N/A	Cancerous cell imaging and long-term monitoring via nucleolus targeting	[170]
Lysosome	2018	CA, urea	hydrothermal	440	37	Lysosome imaging, pH monitoring	[171]
	2019	1,2,4,5-Tetraaminobenzene, dexamethasone	microwave-assisted hydrothermal	568–610	2.01	Formaldehyde detection, lysosome imaging	[172]
	2020	Chloranil and triethylenetetramine	hydrothermal	510	30.8	Lysosome imaging, long-term tracking	[173]
Cell Membrane	2024	Tridecanedioic acid, m-diethylaminophenol	solvothermal	490	14.8	Long-term lipid membrane tracking	[174]

In addition to the use of magnetic hyperthermia for the synthesis of CDs, hybrid systems that integrate magnetic nanoparticles (MNPs) with CDs have also gained increasing attention. These hybrid structures, either in single-particle forms or as colloidally dispersed nanofluids, have shown enhanced functionality in various applications. For instance, MNP-CD hybrids have been employed in dual-modal imaging, combining fluorescence imaging with magnetic resonance imaging (MRI) to improve diagnostic accuracy [175,176]. Additionally, these systems facilitate magnetically guided drug delivery, where an external magnetic field directs MNP-CDs complexes to specific biological targets, enhancing therapeutic efficacy while reducing off-target effects [177]. Furthermore, MNP-CDs composites have been explored in magnetically enhanced photothermal therapy, where the combination of CDs’ photothermal conversion and MNPs’ heat generation under an alternating magnetic field leads to more efficient tumor ablation [178]. These advancements demonstrate the potential of MNP-CDs hybrid systems in next-generation biomedical applications.

### 3.2. Sensors and Detection

**Figure 7:**
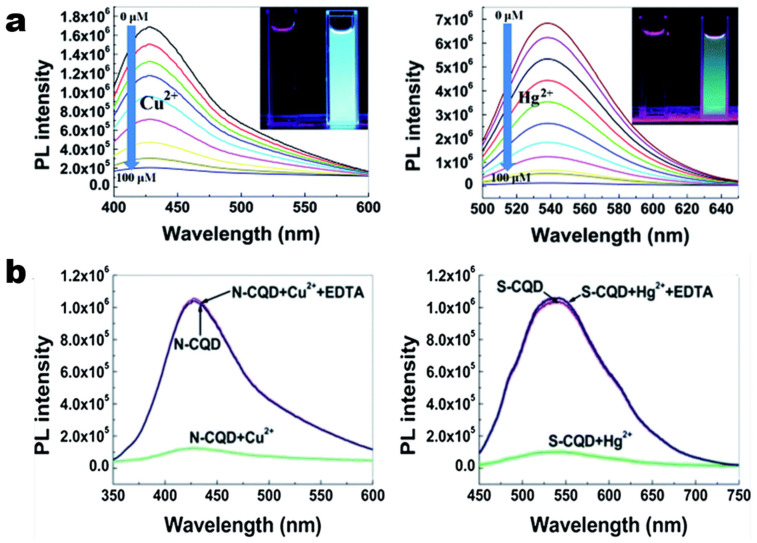
(**a**) The PL spectra versus different Cu^2+^ and Hg^2+^ concentrations. The insets are digital images with and without Cu^2+^/Hg^2+^ addition [132]; (**b**) The PL spectra of N-CQD and S-CQD systems [132].

CDs have found extensive applications in chemical and biological sensing. These nanomaterials exhibit fluorescence changes in response to various analytes, including metal ions, gas molecules, and organic pollutants. CD-based sensors feature high sensitivity, strong selectivity and rapid response speed. Generally, they can be acquired via three distinct design approaches: (1) Direct interaction between CDs and analytes, where fluorescence modulation (e.g., quenching) occurs due to the binding of surface groups; (2) Integration of specific receptors with CDs to realize surface functionalization; and (3) Integration with complementary materials like quenchers or fluorophores to create hybrid sensing platforms [179]. The nitrogen- and sulfur-doped CDs obtained after the oxidation cutting of the carbon nanoparticles previously extracted from ink and subsequent reduction doping treatment have their fluorescence quenched by Cu^2+^ and Hg^2+^, respectively (Figure 7a). After tests, it was discovered that the fluorescence quenching was induced by the coordination between the metal ions and the surface groups of the CDs. After the addition of the chelating agent (EDTA), the fluorescence can be restored (Figure 7b), which suggests its potential for reuse as a fluorescence detection system [132]. Moreover, CDs can also be used for the detection and removal of pollutants in the environment. For instance, the fluorescence of the magnetic CDs fabricated by Song et al. can be quenched by Hg^2+^, and Hg^2+^ in the samples and can be separated and removed for at least three times (with a removal efficiency as high as 98.30%) [180]. This showcases CDs’ dual role in detection and remediation, providing a sustainable approach to addressing heavy metal contamination. Despite these advances, challenges persist in leveraging CDs for sensors and environmental monitoring. Specifically, achieving high sensitivity while ensuring scalable synthesis and maintaining functional stability in complex environments remains critical for transitioning CDs from laboratory research to practical applications.

### 3.3. Optoelectronic Devices

**Figure 8:**
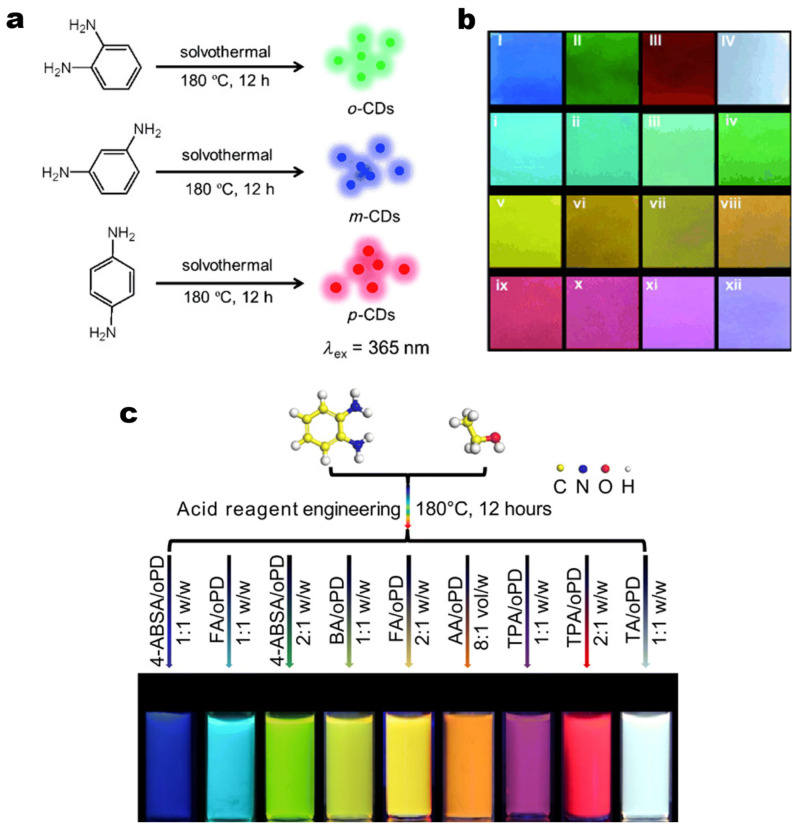
(**a**) Schematic illustration of preparation of three colors CDs [16]; (**b**) Photoluminescence photographs of m-CDs, o-CDs, p-CDs, and their mixtures in PVA composite films UV irradiation [16]; (**c**) Synthetic strategy of full-color fluorescent CDs [181].

Due to their unique optical and electrical properties, CDs show great potential in the field of optoelectronics. CDs possess high specific surface area, high electron mobility, electron acceptor/donor characteristics and excellent photostability, making them ideal candidate materials for LEDs and luminescent solar concentrators (LSCs). For example, in 2015, Jiang et al. utilized o-PDA, m-PDA, and p-PDA as precursors to synthesize a series of CDs that emit red, blue, and green light which are the primary colors, respectively (Figure 8a). By systematically varying the proportions of these CDs, they successfully fabricated full-color- and white light-emitting films (Figure 8b). Notably, the color coordinates of the white film were (0.33, 0.34), closely approximating the standard white light [16]. The successful realization of full-color CDs has significantly spurred interest in exploring the potential of carbon dots as a replacement for traditional LED phosphors. In 2020, Wang et al. utilized o-PDA as the carbon source and introduced varying proportions of functional groups into the structure of CDs using appropriate acids, thereby successfully fabricating full-color CDs with notable fluorescence quantum yields (Figure 8c). Notably, the CDs synthesized using tartaric acid and o-PDA as precursors did not require further proportional mixing or modification and could directly produce WLEDs with photoluminescence covering the entire visible spectrum [181]. In the context of LSCs, Gong’s group pioneered the application of CDs in 2017, demonstrating their high quantum yield, low toxicity, and cost-effectiveness [182]. However, the limited spectral absorption range and significant reabsorption losses highlighted the need for further optimization. Over subsequent years, the team systematically improved their strategies, expanding the absorption range and enhancing photoelectric conversion efficiency [183,184,185]. By 2023, they synthesized yellow-emitting CDs with a remarkable Stokes shift of 193 nm and a quantum yield of 61%, enabling large-area LSCs to achieve a power conversion efficiency of 4.1% and a light conversion efficiency of 4.56% [186]. With negligible reabsorption losses and exceptional stability, these CDs represent a significant breakthrough towards the practical application of LSC technology.

### 3.4. Food Packing

**Figure 9:**
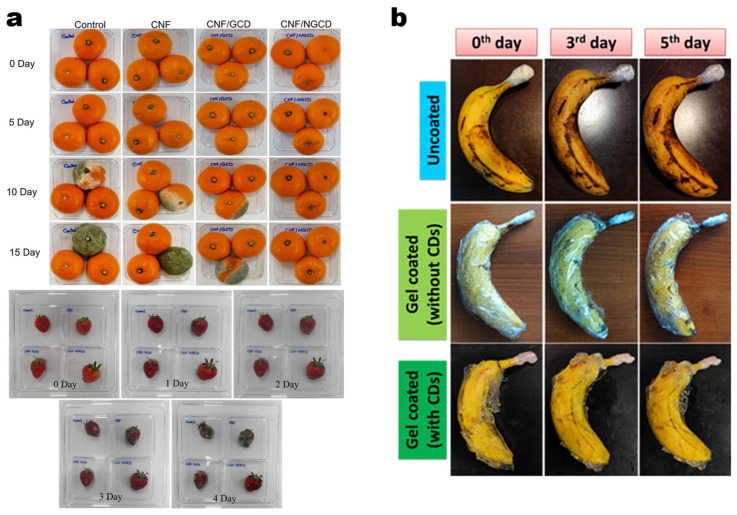
(**a**) Photograph of tangerines’ and strawberries’ appearance change [188]; (**b**) Photograph of bananas on different days [193].

CDs have shown great potential in the field of food packaging, particularly in active, degradable, and smart packaging. Their antioxidant, antibacterial, and fluorescent properties make them ideal candidates for developing innovative food packaging materials (Figure 9a) [187,188,189]. For instance, CD-based biopolymer films, such as thermoplastic starch/κ-carrageenan composites, exhibit excellent mechanical strength, UV resistance, and moisture barrier capabilities, significantly extending the shelf life of food [190]. Additionally, cross-linked gelatin films enriched with CDs demonstrate remarkable antioxidant activity (up to 94.08%) and strong antibacterial effects against *Escherichia coli* and *Staphylococcus aureus*, effectively prolonging the shelf life of chilled fish slices [191]. The adoption of green synthesis techniques for CDs has further propelled their application in degradable and sustainable packaging. CDs derived from food waste and agricultural by-products adhere to green chemistry principles, offering an environmentally friendly alternative to petrochemical plastics [192]. For example, semi-interpenetrating hydrogel films incorporated with CDs exhibit excellent moisture regulation, high elasticity, and biodegradability, making them highly suitable for active and intelligent packaging systems (Figure 9b) [193]. Furthermore, the unique fluorescence properties of CDs position them as ideal materials for smart packaging, enabling real-time monitoring of food quality through detection of pH variations or oxidation by-products, thus facilitating dynamic freshness assessments [190,191]. Despite these advancements, the practical application of CDs in food packaging still faces several challenges. Future research should focus on integrating multifunctionality—combining antibacterial, antioxidant, and intelligent detection capabilities—to improve the overall performance of packaging systems.

### 3.5. Energy Application

CDs have emerged as highly promising materials for energy storage technologies, including lithium-ion batteries (LIBs), sodium-ion batteries (SIBs), and supercapacitors. These materials, with their remarkable properties such as high surface-to-volume ratio, tunability in surface chemistry, superior electron mobility, and intrinsic conductivity, offer an excellent platform for enhancing energy storage performance [194]. Specifically, their surface functionalization allows for the fine-tuning of charge transfer characteristics, enabling them to act as effective conductive additives and electrode modifiers [195]. These modifications contribute to the enhancement of charge transfer efficiency, energy density, and cycle stability in energy storage devices, making them competitive with traditional materials used in LIBs and SIBs [196,197,198].

Recent studies have focused on doping CDs with heteroatoms, which has been shown to significantly improve the electrochemical performance of batteries and supercapacitors by boosting charge transfer kinetics and capacitance. For example, nitrogen-doped CDs exhibit a remarkable increase in capacitance due to the nitrogen-induced charge delocalization and enhanced electron conductivity [199,200]. Similarly, sulfur-doped CDs have been proven to enhance charge retention and electrochemical stability, making them ideal for applications in both supercapacitors and batteries [201,202,203]. In supercapacitors, CDs have demonstrated exceptional performance, not only in increasing charge storage capacity but also in reducing internal resistance, a critical factor in improving energy density and extending cycle life. For example, N, S co-doped CDs have been shown to facilitate faster ion diffusion, minimize energy loss, and ensure efficient charge/discharge cycles, which are essential for long-term device performance [201,204].

This versatility positions CDs as key players in next-generation energy storage technologies. Their role in high-efficiency supercapacitors, long-cycle next-generation batteries, and hybrid systems integrating graphene and carbon nanotubes represents a significant advancement in energy storage research. The scalable production of these nanomaterials could provide economically viable and environmentally friendly solutions for large-scale energy storage systems, ranging from electric vehicles to renewable energy storage applications.

## 4. Key Challenges in Large-Scale Synthesis of CDs

While CDs have demonstrated remarkable potential in biomedicine, optoelectronics, sensors, food packaging and energy applications, their widespread commercialization remains hindered by challenges in large-scale synthesis. The transition from laboratory-scale methods to industrial production faces critical barriers, including yield consistency, energy efficiency, purification complexity, economic feasibility, and regulatory compliance. Addressing these issues is essential to facilitate the industrial adoption of CDs.

### 4.1. Yield and Scalability Challenges

Most reported CDs synthesis methods are developed at small scales (mg to g level), ensuring precise control over reaction conditions. However, large-scale production (kg to ton level) demands batch-to-batch consistency, which is often difficult to achieve due to heat and mass transfer constraints. Hydrothermal and solvothermal approaches, despite their widespread use, suffer from poor scalability, leading to variations in product quality.

To address these challenges, continuous-flow synthesis has emerged as a promising strategy to improve reaction uniformity and process control. Unlike conventional batch systems, continuous-flow reactors provide better temperature and concentration gradients, allowing for enhanced mass and heat transfer while minimizing batch-to-batch variability [205]. Microfluidic reactors and flow chemistry systems, in particular, enable precise reaction tuning and have demonstrated the ability to produce CDs with uniform particle size and tunable optical properties at an accelerated rate, making them viable for large-scale manufacturing [206,207,208,209,210]. Integrating continuous-flow techniques with real-time monitoring systems could further optimize reaction efficiency and enhance production reproducibility. Moreover, optimized microwave-assisted synthesis has shown potential in maintaining consistent heating across larger batches [211]. By fine-tuning reaction parameters, such as power input and exposure time, microwave synthesis can improve uniformity in carbonization. Additionally, enhanced stirring efficiency and precise temperature regulation can mitigate aggregation issues, ensuring homogenous carbonization and further facilitating industrial scalability.

The stability of CDs is one of the most critical factors influencing their performance in various applications, particularly in biomedical and optoelectronic fields. The stability of CDs is affected by factors such as size, surface characteristics, and the dispersion medium. While much research has been focused on the photophysical and chemical stability of CDs, colloidal stability has also garnered significant attention in recent years due to its impact on aggregation and optical properties. Poor colloidal stability can lead to aggregation, resulting in fluorescence quenching, reduced bioavailability, and even increased cytotoxicity, which may negatively affect their performance in sensitive applications such as bioimaging and drug delivery. Currently, Dynamic Light Scattering (DLS) is widely used to monitor nanoparticle aggregation and colloidal behavior, providing real-time insights into particle size distribution and stability mechanisms [212]. This technique offers valuable guidance for the large-scale production of uniform and stable CDs.

### 4.2. High Energy Consumption and Long Reaction Time

Hydrothermal and solvothermal synthesis often require prolonged reaction times (6–24 h) at elevated temperatures (120–250 °C), significantly increasing energy consumption and production costs. For large-scale applications, these long processing times pose a serious limitation to continuous manufacturing. The adoption of rapid synthesis techniques, such as microwave, plasma-assisted, and magnetic hyperthermia, could substantially reduce reaction time while maintaining high product yields. Additionally, low-temperature synthesis approaches utilizing molten salts or catalytic activation could decrease energy demand [213], making large-scale production more economically viable. Enhancing reaction kinetics through functional catalysts may further improve the efficiency of CDs synthesis by accelerating carbonization under milder conditions.

### 4.3. Purification and Quality Control Issues

Ensuring high purity in large-scale CDs production remains a formidable challenge [214,215], as traditional purification techniques like dialysis [216], centrifugation [217], and chromatography [218], while effective in laboratory settings, are often impractical due to their time-intensive nature and high operational costs [219]. High-performance chromatography offers superior resolution and shorter elution times, yet its frequent maintenance and operational expenses hinder industrial adoption [220]. Alternative strategies, such as membrane filtration and centrifugation, provide cost-effective, high-throughput purification solutions. In particular, gradient centrifugation has been employed to selectively remove undesired particles while preserving the final product yield [220].

However, large-scale purification presents an inherent trade-off between purity and yield. While rigorous purification processes effectively eliminate unwanted by-products and improve biocompatibility, excessive purification cycles can significantly reduce final product yields, making large-scale production economically unfeasible. This issue is particularly critical for biomedical applications such as cellular imaging and phototherapy, where residual impurities can lead to fluorescence quenching, increased cytotoxicity, and loss of biofunctionality. To address this challenge, solvent precipitation has emerged as a promising large-scale purification approach. Unlike conventional dialysis or chromatography, which are often time-consuming and result in significant material loss, solvent precipitation leverages selective solubility differences to efficiently remove residual precursors and unreacted by-products. Recent studies have demonstrated its effectiveness in improving the purity of CDs while maintaining high product yields. For instance, ethanol precipitation has been successfully applied to nitrogen-doped CDs (NCQDs) derived from chitosan, efficiently eliminating unwanted impurities and yielding highly purified NCQDs suitable for biological applications [221]. Similarly, a unique self-precipitation phenomenon observed in red-emitting CDs has provided an alternative route for obtaining solid-phase high-purity CDs without extensive post-processing [222]. These findings suggest that solvent precipitation techniques could serve as scalable, cost-effective solutions for achieving high-purity CDs with minimal production losses, thereby facilitating their industrial application.

Furthermore, automated quality control systems [205]—incorporating real-time spectroscopy, machine learning-driven defect detection, and continuous monitoring [223]—are essential for ensuring batch-to-batch reproducibility and maintaining consistent physicochemical properties of CDs in large-scale production. With the increasing application of machine learning in carbon dot synthesis [224,225], leveraging real-time impurity analysis to dynamically adjust purification parameters presents a promising research avenue. Machine learning-assisted purification strategies could facilitate the development of scalable methods that ensure high product purity while minimizing material loss, offering a practical balance between efficiency and yield in large-scale production.

### 4.4. Economic and Environmental Concerns

Beyond technical challenges, economic and environmental factors play a crucial role in determining the feasibility of large-scale CDs production. The reliance on high-purity chemical precursors, such as o-PDA and CA, significantly increases production costs, while the use of organic solvents raises concerns regarding environmental sustainability. A promising approach to addressing these issues involves the utilization of biomass-derived carbon sources, including food waste, agricultural residues, and industrial by-products, which not only lower material costs but also align with green chemistry principles. Furthermore, minimizing solvent usage through the development of water-based or solvent-free synthesis techniques can help reduce production expenses while mitigating environmental risks.

### 4.5. Structural Ambiguity and Property Control

Despite extensive research, the precise structural composition of CDs remains ambiguous, which complicates the establishment of a definitive structure–property relationship. CDs are generally considered to possess a sp^2^/sp^3^-hybridized carbon core with various surface functional groups, yet the exact atomic arrangement and bonding nature vary significantly depending on synthesis conditions and precursors [226,227]. This structural uncertainty presents major challenges for large-scale production, particularly in ensuring batch-to-batch consistency, precise control over optoelectronic properties, and reproducibility of functional groups. Inconsistent structural features may lead to variations in fluorescence quantum yield, solubility, and stability, ultimately affecting industrial applicability. Moreover, the lack of standardized characterization techniques further complicates quality control in large-scale production. While high-resolution techniques such as solid-state NMR, X-ray absorption fine structure, and mass spectrometry have provided insights into CD structures [228,229], these methods are not readily adaptable for large-scale, high-throughput characterization. To bridge this gap, real-time monitoring techniques such as Raman spectroscopy, dynamic light scattering, and real-time fluorescence spectroscopy are being explored as potential solutions for inline quality control. Developing efficient, scalable characterization approaches will be essential for achieving reliable and reproducible CDs production at an industrial scale.

## 5. Conclusions and Outlook

CDs have demonstrated extensive application potential in fields such as biomedicine, optoelectronics, and smart food packaging with their exceptional optical properties, low toxicity, good biocompatibility, and cost-effectiveness. This article systematically analyzes the application prospects of CDs in imaging, drug delivery, photovoltaic devices, and smart packaging, while emphasizing the latest advancements in large-scale synthesis technologies. The development of scalable synthesis methods is progressively steering CDs toward industrial production, with numerous efforts focused on reducing equipment requirements and enhancing process efficiency. Various synthesis methods—including hydrothermal, solvothermal, microwave, pyrolysis, and solid-state carbonization—have been significantly optimized to improve yield, reduce energy consumption, and achieve environmentally friendly production. For example, the microwave method, known for its rapid and efficient characteristics, has emerged as a promising solution for industrial-scale production. Similarly, pyrolysis and solid-state carbonization methods, characterized by their simplicity and eco-friendly processes, are gaining traction for large-scale applications. By optimizing doping elements, precursor selection, and reaction conditions, researchers have achieved notable improvements in the optical properties, stability, and functional characteristics of CDs, establishing a robust foundation for their practical implementation at scale.

Despite these advances, the large-scale synthesis and practical application of CDs continue to face several challenges. First, in industrial production, the development of more economical, efficient, and environmentally friendly synthesis methods is crucial to meet the growing demand for scalable CDs. Hydrothermal and solvothermal methods are among the most widely reported approaches; however, they often involve high energy consumption, costly equipment, and prolonged reaction times. The extended duration and significant energy requirements make them unsuitable for continuous industrial production. Emerging strategies, such as biomass-derived CDs or solid-state carbonization, have demonstrated potential for achieving lower costs and reduced environmental impact. Second, the diverse application scenarios of CDs—ranging from biomedicine and optoelectronics to smart packaging—require precise customization of their characteristics to fulfill specific performance demands. For instance, biomedical applications prioritize biocompatibility and stability, while optoelectronics focus on optical tunability and quantum yield. However, balancing the increasing complexity of multifunctional customization with the scalability and cost-efficiency of synthesis remains a key challenge. Third, translating laboratory-scale optimizations into industrial-scale processes poses significant challenges, particularly in adapting synthesis techniques to large-scale production. This transition necessitates innovations in equipment design, process control, and quality management to ensure consistency and scalability. Many conventional synthesis routes, such as hydrothermal and solvothermal methods, rely on high-temperature and high-pressure conditions, which not only increase operational complexity but also impose stringent requirements on industrial equipment. Addressing these technical demands will be crucial for the practical implementation of CDs in large-scale manufacturing.

Additionally, the purification process, critical to ensuring product quality, poses challenges when scaled up. Effectively purifying large quantities of CDs within a short timeframe and at low cost, while minimizing polluted water generation in post-treatment, is a key obstacle for green production. Furthermore, the long-term stability and safety of CDs in complex environments require rigorous verification to meet practical application standards. Although most studies have demonstrated that CDs are non-toxic within appropriate concentration ranges (>80% cell viability), their long-term pathways and metabolic fate in complex biological environments remain poorly understood, raising concerns for clinical feasibility. Future research should prioritize these aspects to establish safety benchmarks. Moreover, by-products and residues generated from different synthesis methods may pose risks to biocompatibility, necessitating further optimization of cleaning and purification processes, particularly in large-scale production.

## Data Availability

The data presented in this study are available on request from the corresponding author.

## Abbreviations

The following abbreviations are used in this manuscript:

CDs	Carbon Dots
GQDs	Graphene Quantum Dots
CQDs	Carbon Quantum Dots
CNDs	Carbon Nano Dots
LED	Light-emitting Diode
WLED	White Light emitting Diode
PA	Phosphoric Acid
EDA	Ethylenediamine
RTP	Room Temperature Phosphorescence
PD	Phenylenediamine
o-PDA	o-Phenylenediamine
m-PDA	m-Phenylenediamine
p-PDA	p-Phenylenediamine
CA	Citric Acid
DMF	N,N-dimethylformamide
BA	Boric Acid
DETA	Diethylenetriamine
AA	Acrylic Acid
CJ	Calamansi juice
NIR	Near Infrared
PMDA	1,2,4,5-Benzenetetracarboxylic Anhydride
ISC	Intersystem Crossing
MHT	Magnetic Hyperthermia Method
TTDDA	4,7,10-Trioxa-1,13-tridecanediamine
PEG	Polyethylene Glycol
CRI	Color Render Index
N/A	Not Available
RT	Room Temperature
DNA	Deoxyribo Nucleic Acid
MNP	Magnetic Nanoparticle
LIB	Lithium-ion Batterie
SIB	Sodium-ion Batterie
PTCDA	Perylene Tetracarboxylic Anhydride
PEI	Polyethylenimine
Si-QAC	Dimethyloctadecyl [3-(trimethoxysilyl)propy]ammonium Chloride
LSCs	Luminescent Solar Concentrators
PVA	Poly(vinyl alcohol)
UV	Ultraviolet
